# Level and Associated Factors of Knowledge regarding Menstrual Hygiene among School-Going Adolescent Girls in Dang District, Nepal

**DOI:** 10.1155/2020/8872119

**Published:** 2020-10-21

**Authors:** Chet Kant Bhusal, Sigma Bhattarai, Raju Kafle, Rubina Shrestha, Pradip Chhetri, Kishor Adhikari

**Affiliations:** ^1^Department of Community Medicine, Universal College of Medical Science and Teaching Hospital, Tribhuvan University, Rupandehi, Bhairahawa, Nepal; ^2^Universal College of Nursing Science and Teaching Hospital, Tribhuvan University, Rupandehi, Bhairahawa, Nepal; ^3^Department of Pediatrics, Universal College of Medical Science and Teaching Hospital, Tribhuvan University, Rupandehi, Bhairahawa, Nepal; ^4^School of Public Health and Department of Community Medicine, Chitwan Medical College and Teaching Hospital, Tribhuvan University, Bharatpur, Nepal

## Abstract

**Background:**

Adolescent girls in developing countries do not have proper information, and proper information is covered up by sociocultural boundaries resulting in various morbidities. This study aimed to determine level of knowledge and its associated factors regarding menstrual hygiene amongst adolescent school girls in Dang district, Nepal.

**Methods:**

Institution-based cross-sectional study was conducted between April and October 2019 among 406 adolescent girls studying in grades 8–10 between ages of 10–19 years in Dang district, Nepal. From a total of 10 local units, 5 were selected randomly. Out of the selected 5 units, 10 schools consisting of 5 government and 5 private schools were selected through disproportionate stratified random sampling. A further 406 students were then selected randomly from the 10 selected schools. Bivariate analysis was used primarily to assess the association between dependent and independent variables. Variables which were associated with bivariate analysis were entered into a multivariable logistic regression model to identify associated factors of menstrual hygiene practice.

**Results:**

The mean age and family size were 15.13 ± 1.19 and 5.58 ± 1.81, respectively. A total of 87.7% of adolescents had good knowledge regarding menstrual hygiene. Adolescents living in rural area (AOR = 0.27, CI: 0.12–0.61, *p* ≤ 0.001), private schools (AOR = 6.10, CI: 1.58–23.46, *p* ≤ 0.001), mothers who can read and write (AOR = 0.22, CI: 0.07–0.64, *p* ≤ 0.001), fathers who have up-to-grade-10 education (AOR = 5.15, CI: 1.84–14.39, *p* ≤ 0.001), and living only with mothers (AOR = 0.29, CI: 0.12–0.69, *p* ≤ 0.018) were significantly associated with level of knowledge of menstrual hygiene.

**Conclusions:**

Though the majority of respondents had a good level of knowledge regarding menstrual hygiene, there was a knowledge gap in specific areas. The level of knowledge was significantly poor among adolescents in rural areas and those living only with mothers. Thus, this study concerns the need for policy makers to focus on specific education regarding menstrual hygiene in rural areas including both parents.

## 1. Introduction

Menstruation is a normal phenomenon among grown females who experience flaking of blood for one to seven days every month from the age of menarche to menopause [1]. Girls living in low-middle income countries have inadequate knowledge and understanding regarding menstruation prior to their menarche [2]. Due to lack of previous information regarding menstruation, girls have special feelings such as fear, embarrassment, and guilt during their cycle [3]. The inadequate knowledge of menstrual hygiene may lead to unhygienic practice which increases vulnerability to different health outcomes, school drop-out, poor academic performance, and ultimately resulting in poor quality of life [4]. Menstrual hygiene is a necessary element in every woman's life [5]. Menstrual hygiene deals with special healthcare requirements and necessities such as the use of sanitary pads or clean and soft absorbent, adequate washing of the genital area, and proper disposal of used absorbent for women during their menstruation [6, 7].

Due to little knowledge about menstruation and menstrual hygiene, millions of women throughout the world are unable to manage their menstrual periods. This results in an increased risk of having different complications such as reproductive tract infections and pelvic inflammatory and urinary tract diseases [8, 9]. Several studies conducted in schools throughout parts of Nepal and India show inadequate knowledge and practices of menstrual hygiene [10–12]. Women and young girls in low-income countries do not have proper awareness about hygienic practices; hence, they lack suitable absorbent during their menstruation [13–15]. A study conducted in Nagpur found more than three-fourths of the girls were not aware of the cause and source of bleeding [12]. Adolescent girls especially in rural and tribal communities do face many complications to obtain the right kind of information even from their parents due to social and cultural stigma and exclusions [16, 17]. Due to these taboos and sociocultural and religious restrictions, girls remain unaware of scientific facts and hygienic practice that may cause harmful health outcomes [9, 18]. Even though menstruation is a natural progression, it is linked with misconceptions, mismanagements, and challenges among girls in developing countries [19]. Many studies reported that poor management of menstrual period may accompany discomfort, reproductive tract infection, smelling, and embarrassment which further leads to various sexually transmitted diseases [9, 16, 20–22]. Lack of adequate privacy and sanitation for school girls proved to be an issue, making them vulnerable to mental, emotional, and physical problems especially during their menstruating days [22]. Social taboos and misconceptions on the subject of menstruating girls and menstrual hygiene develop in gender inequality and degradation of women empowerment [23]. Though menstruation is a normal body function for females, handling it is considered a major challenge for every adolescent girl [24]. A previous study done in rural Nepal highlighted that 70.7% of girls answered they should not go to school during menstruation [25]. Women who have good knowledge about menstrual hygiene will also have good practice; thus, they will be less vulnerable to reproductive tract infection and its consequences [9].

Although literature on various aspects of menstrual hygiene is available to young girls, few studies are carried out to investigate the associated factors of knowledge regarding menstrual hygiene [4]. As a result of this, the present study aims to determine different factors associated with knowledge level regarding menstruation hygiene among school-going adolescent girls in Dang district, Nepal.

## 2. Materials and Methods

### 2.1. Study Design and Source of Population

Institution-based cross-sectional study was carried out in Dang district, Nepal, among young adolescent girls, between the ages of 10–19, during April 2019-October 2019. School-going adolescent girls from both government and private schools studying in grades 8, 9, and 10 of selected schools were included in the study [26]. Girls in the same class whose menarche was not started till the day of data collection and who showed previous mental problems and who were not present during the day of data collection were excluded from the study [26].

### 2.2. Sample Size Determination and Sampling Technique

**Figure 1:**
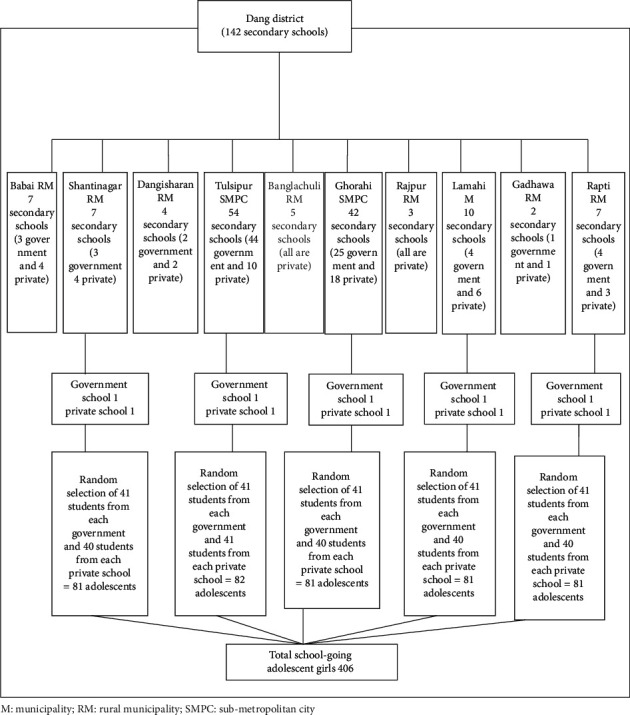
Multistage stratified probability random sampling technique.

Sample size was 406 which was calculated using formula *N* = Z^2^pq/L^2^ [27] with 95% level of confidence interval, critical value *Z* = 1.96, and 6% margin of error, and 40.6% of rural adolescent girls of Nepal had good knowledge of menstrual hygiene [25]. Since multistage stratified probability random sampling was used as a sampling technique, the initial sample size of 258 was multiplied by the design effect 1.5; hence, *n*=258*∗*1.5=387. Further by adding 5% nonresponse rates, the final sample size of the study is 406. A multistage probability random sampling among a total of 142 secondary schools of Dang district was used. Among the total of 10 local units, 5 were selected randomly. Then, from the selected 5 units, 10 schools consisting of 5 government and 5 private schools were selected by disproportionate stratified random sampling technique through a non-replacement lottery method. Furthermore, 406 students were selected at random from the 10 chosen schools which consist of 41 students from each government and 40 students from each private school of Dang district, Nepal (Figure 1). Since the sample size of the study was 406, further one additional dataset was taken from a private school.

### 2.3. Data Collection Procedures and Validity

Data was collected using pretested semi-structured questionnaire by applying self-administered interview technique. The questionnaire was translated into Nepali and then retranslated into English language to identify misinterpretations. The questionnaire was pretested among 10% of total sample size residing in Bhairahawa, Rupandehi. Both English and Nepali version questionnaires were made and used according to familiarity of students. Four data collectors were involved in collecting data, including one principle investigator with Master's degree in Public Health as well as Master's in Sociology and three trained enumerators with Master's qualifications in Information Communication Technology, Bachelor in Public Health, and Bachelor of Science in Agriculture.

### 2.4. Data Processing and Analysis

Data were entered into Microsoft Excel and exported to Statistical Package for the Social Sciences (SPSS) software version 20 for analysis. Simple descriptive statistics such as frequencies, means, and standard deviations were calculated and associated factors between the different variables in relation to the outcome variable were measured by chi-square test having odds ratio with 95% confidence interval. Primarily, bivariate analysis was used to check whether the variables had an association with the dependent variable individually. Multivariate logistic regression was conducted to analyze factors which were associated with level of knowledge regarding menstrual hygiene. All variables found to be associated with main outcome variables in bivariate analysis (*p* < 0.05) were entered into the multivariate logistic regression model to find associated factors of knowledge of menstrual hygiene. The goodness of fit of multivariate logistic regression was checked by using Nagelkerke R Square and Variation Inflation Factor (VIF). The result showed that Nagelkerke R square was 0.366; it means that 36.6% variation in knowledge level regarding menstrual hygiene was explained by the significant independent variables. Similarly, VIF of all significant independent variables lies in the range of 1-2 so there is no multicollinearity among independent variables. After checking this, all multivariate logistic regression was applied to find the net effect of independent variables in knowledge level regarding menstrual hygiene. The data was summarized and adjusted odds ratios (AORs) were estimated and their corresponding value at 95% confidence intervals was computed.

### 2.5. Setting

Dang district is located in inner Terai and midhills of Rapti zone in the mid-western development region of Nepal. The district consists of 2 submetropolitan cities and 1 municipality as well as 7 rural municipalities and 3 electoral consistencies. According to Central Bureau of Statistics 2011, the district has a total population of 552,583. The annual population growth rate of the district is 1.78 [28]. In total, there are 142 secondary schools including 86 private and 56 government schools; similarly, there is a total of 24,622 students studying at secondary school level in Dang district which includes 12,905 female and 11,717 male students [29].

### 2.6. Measurement of Knowledge on Menstrual Hygiene

Students' knowledge of menstruation and its hygienic management was recorded using a scoring system adopted from a past study [19]. Students' knowledge level regarding menstrual hygiene was scored through 12 knowledge-specific questions. Each “correct response” earned one point, whereas any “incorrect” or “don't know response” got zero. The sum score of knowledge was calculated out of 12 points. Respondents who scored 0–6 points were considered as having poor knowledge, whereas participants who scored 7–12 points were considered as having good knowledge.

### 2.7. Ethics Approval and Consent to Participate

Ethical approval was obtained from of Universal College of Medical Science and Teaching Hospital Institutional Review Committee (UCMS/IRC/063/19). Concerned stakeholders were officially contacted with letters and permission was obtained from all levels. The study was explained to participants. Since most of the respondents of this study were below the age group of 18 years, verbal consent was taken from each child's parent or guardian through telephone. After informing their parents, written informed consent was also taken from school teachers before interview.

## 3. Results

The mean age and mean family size were 15.13 ± 1.19 and 5.58 ± 1.81, respectively. From a total of 406 adolescent girls, more than half (55.7%) lived in submetropolitan city and municipalities. About one-fourth (23.6%) of the respondents' mothers were unable to read and write, with 5.2% of respondents' mothers having a Bachelor degree and further education. Slightly less than half (47.5%) of the mothers were homemakers. More than one-fourth (27.6%) of the respondents' fathers were engaged in small-scale business operations (Table 1).

The majority (89.4%) of the girls had heard about menstruation before they had their menarche, where the foremost source of information was sisters or mothers at 71.4%, followed by friends or relatives at 36.0%, teachers or books at 31.8%, and radios, televisions, or newspapers at 12.6%.  Table 2 presents the adolescent school girls' knowledge about menstruation and its management. More than four-fifths (84.5%) of participants knew menstruation is a cyclic process, and 88.7% knew the common age range of menarche. The majority of girls at 90.9% knew about absorbents during menstruation. Among the total, 82.5% and 84% of adolescent girls were aware that poor menstrual management causes infection and personal hygiene helps in pain management, respectively. Less than one-third at 31.5% knew the common age range of menopause (Table 2).

The mean score of school girls' knowledge of menstruation and its hygienic management was 9.23 ± 2.11. The study revealed that the majority (87.7%) had good knowledge about menstruation (Table 3).

Table 4 presents the multiple regression analysis of knowledge level of menstrual hygiene with associated factors. Those variables which were found statistically significant with *p* value less than or equal to 0.05 in bivariate analysis were entered into the multivariate regression analysis model which identified residence of living, type of school, mother's education, father's education, and living status in family as associated factors with knowledge of menstrual hygiene. Adolescent girls who were living in rural areas were found 0.27 times less likely (AOR = 0.27, CI: 0.12–0.61) to have good knowledge of menstrual hygiene. Similarly, odds of having good knowledge of menstrual hygiene with students studying in private schools were 6.10 times more likely (AOR = 6.10, CI: 1.58–23.46). Girls from literate mothers, i.e., who can read and write, were 0.22 times less likely (AOR = 0.22, CI: 0.07–0.64) to have good knowledge regarding menstrual hygiene and its management. However, for father's education, those who had completed primary to secondary (up to grade 10) were 5.15 times more likely (AOR = 5.15, CI: 1.84–14.39) to have good knowledge regarding menstrual hygiene and its management than their counterparts who were from illiterate backgrounds. Adolescent school girls who were living only with mothers were 0.29 times less likely (AOR = 0.29, CI: 0.12–0.69) to have good knowledge regarding menstrual hygiene and its management than those who were living with both of their parents. After subjecting to multivariate model, religion, size of family, father's occupation, and family earning status were found confounders for level of knowledge (Table 4).

## 4. Discussion

Residence, types of school, mother's education, father's education, and living with both parents were associated with knowledge level of menstrual hygiene in this study. Less than one-third of adolescent girls were aware about the age range of menopause. The finding of this study revealed that the majority (87.7%) of school-going adolescent girls had good knowledge about menstruation and its management which is in line with the study conducted in Amhara regional state, Ethiopia [30], and northwest Nigeria [31]. The study done in southwestern Nigeria and in Sokoto, Nigeria, found slightly lower proportion of the respondents had high knowledge, respectively [32, 33]. In contrast to this study, the study done in Nepal [25, 34], Bangladesh [35], Baghdad, Iraq [36], Northern Ethiopia [19], and Nigeria [37, 38] revealed lower portion of school adolescent girls had good knowledge about menstruation. This difference might be due to change in time and progression of educational supplies as compared to previous time as well as because of divergence scoring system for measuring the knowledge level of menstrual hygiene in different studies.

The present study showed that the majority of the girls heard about menstruation before they had menarche which is in line with the study conducted in northeast Ethiopia and in southwestern Nigeria [19, 33]. However, a study done in India found slightly lesser than two-thirds of adolescent girls were aware about menses before menarche [39, 40]. In contrast to this study, other studies done in Ranchi, India [41], and Nagpur district of India [12] found less than half of adolescent girls had ideas prior to attaining menarche. This difference might be due to the fact that women from least developed and developing countries might not express their views to educate their daughters due to taboo and myth regarding menstruation. The foremost source of information in this study was mothers and sisters which is in agreement with other studies from different low-income countries, such as rural Nepal [11], India [12, 39, 41], Northeast Ethiopia [19], and Nigeria [37]. The majority of the school-going adolescent girls in this study knew menstruation is a cyclic process which is in line with study conducted in northwestern Nigeria [31]. Most of school-going adolescent girls in this study were aware that menstruation is a normal physiological process which is in line with the study done in remote Doti district [34] and Chitwan district [42] of Nepal. However, studies conducted in India [41] and Nigeria [37] found lower proportion of school girls reported that it is a physiological process. This difference might be due to the different study setting as studies conducted in Nepal showed similar results.

The majority of the girls in this study knew the common age range of menarche which is in line with a study conducted in northeast Ethiopia [19]. More than three-fourths of the girls in this study knew about the correct definition of menstruation which is in line with the study conducted in Nigeria [31]. In the current study, the majority of the adolescent girls knew the duration of a normal menstrual cycle which is in line with the studies done in Nepal [43] and northeast Ethiopia [19]; however, slightly less than two-thirds knew the correct length of cycle in the study done by Houston et al. [44]. This difference might be due to progress in educational supplies with advancement of time. More than half of the girls in this study were aware that menstruation is due to hormones; however, studies conducted in rural Nepal and Nigeria found lower proportion of girls were aware [25, 37]. This difference might be due to change in time and different study setting. In the present study, slightly less than three-fourths of girls knew normal menstrual bleeding duration which is supported by the study conducted in tribal Gujjar, India [40]; however, another study conducted in Bangladesh found more than three-fourths of girls correctly knew about normal menstrual bleeding duration [35]. This might be due to different study setting. The present study revealed that four-fifths of school girls knew the correct definition of menstruation cycle which is in line with the study done in northwestern Nigeria [31]. The majority of the girls in the current study knew about some of the absorbents during menstruation which is in line with the study conducted in northwestern Nigeria [31]. More than four-fifths of respondents in this study were aware that poor menstrual management causes infection which is in line with the study done in Ethiopia [45]. Among the total, 84% of adolescent girls in the current study were aware that personal hygiene helps in pain management; however, a study conducted in Nigeria [31] found lower proportion of the girls knew about it. This difference might be due to advancement of time. In the present study, less than one-third of respondents knew the common age range of menopause; this finding is supported by the study done in northwestern Nigeria [31]. More than three-fourths of the adolescent girls in this study said the source of menstrual blood is from the vagina; however, a study done in Nigeria found less than half of adolescent girls knew about the source of menstrual blood [37]. This diversity might be due to different study setting.

In the current study, statistical association was established between mother's education and knowledge level regarding menstrual hygiene which is in line with studies conducted in Nigeria, West Bengal of India, and Ethiopia [30, 37, 46–48]. The study found significant association of knowledge of menstrual hygiene with father's education where adolescent's father who had education up to grade 10 is 5.15 times more likely to have good knowledge. This observation is in agreement with another study from Nigeria [37]. The present study revealed that a person living in rural areas (rural municipalities) was 0.27 times less likely to have good knowledge regarding menstrual hygiene in multivariate analysis. This observation is in agreement with another study done in Ethiopia [47]. Consistently, a study conducted in Amhara province, Ethiopia, found urban girls were 1.80 times more likely to have good knowledge regarding menstrual hygiene [30]. In this study, girls who were studying in private schools were 6.10 times more likely to have good knowledge in comparison to the girls studying in government schools but the study contradicts a study conducted in northwestern Nigeria where type of school was found insignificant regarding knowledge of menstruation [31]. Regarding the living status of adolescent girls, girls who were living with only mothers were 0.50 times and relatives were 0.78 times less likely to have good knowledge of menstrual hygiene compared to living with both parents which is supported by the study conducted in Nigeria [37].

## 5. Conclusions

The majority of the girls had heard about menstruation before they had menarche where the foremost sources of information were sisters or mothers. Even though the majority of respondents had good knowledge regarding menstrual hygiene, there was a knowledge gap in specific areas; for example, few girls knew the common age range of menopause and were less were aware that menstruation is due to hormones. Knowledge level of menstrual hygiene was significantly poor among adolescents in rural areas and those living only with mothers. It is also predisposed by types of schools and mothers' and fathers' education. In this context, this study concerns the need of specific education regarding menstrual hygiene which should be given in rural areas including both parents.

## Figures and Tables

**Table 1:** Distribution of background related characteristics of study population.

General characteristics	Frequency (*n* = 406)	Percentage
Age		
10–13 years	34	8.4
14–16 years	328	80.8
17–19 years	44	10.8
Mean age ± SD; 15.13 ± 1.19		

Residence		
Urban (submetropolitan city and municipalities)	226	55.7
Rural (rural municipalities)	180	44.3

Religion		
Hindu	372	91.6
Non-Hindu (Christian and Muslim)	34	8.4

Family size		
1 to 4	120	29.6
5 and above	286	70.4
Mean family members ± SD; 5.58 ± 1.81		

Type of school		
Government	205	50.5
Private	201	49.5

Education of mother		
Illiterate	96	23.6
Read and write	65	16.0
Primary (1–5)	65	16.0
Secondary (6–10)	101	24.9
SLC and intermediate	58	14.3
Bachelor and above	21	5.2

Occupation of mother		
Homemaker	193	47.5
Agriculture	72	17.7
Business	74	18.2
Government and private	28	6.9
Wage labor	39	9.6

Occupation of father		
Agriculture	88	21.7
Small-scale business	112	27.6
Service (government and private)	46	11.3
Wage labor	52	12.8
Foreign labor	108	26.6

Earning status in family	—	—
Yes	358	88.2

**Table 2:** Adolescent school girls' knowledge about menstruation and its management.

Knowledge about menstruation and its management	Number (*n* = 406)	Percentage
Knew menstruation is a cyclic process	343	84.5
Was aware that menstruation is a normal physiological process	359	88.4
Knew the common age range of menarche	360	88.7
Knew normal menstrual bleeding duration	292	71.9
Knew about definition of menstruation	324	79.8
Knew duration of a normal menstrual cycle	346	85.2
Knew about absorbents during menstruation	369	90.9
Aware that poor menstrual management causes infection	335	82.5
Aware that personal hygiene helps in pain management	341	84.0
Knew the common age range of menopause	128	31.5
Aware that menstruation is due to hormones	233	57.4
Aware that menstruation blood flows from vagina	313	77.1

**Table 3:** Adolescent school girls' knowledge grading on menstruation and its management.

Characteristics	Frequency (*n* = 406)	Percentage
Poor knowledge level (0–6 points)	50	12.3

Good knowledge level (7–12 points)	356	87.7

Total	406	100.0

**Table 4:** Factors associated with level of knowledge in bivariate and multivariate analysis.

Characteristics	Knowledge level (%)		*p* value	^a^COR 95% CI	^b^AOR 95% CI
	Poor knowledge	Good knowledge			
Residence					
Urban	12(5.3)	214(94.7)	—	1	1
Rural	38(21.1)	142(78.9)	<0.001^*∗*^	0.21(0.11–0.42)	0.27 (0.12–0.61)

Religion					
Hindu	42 (11.3)	330(88.7)	—	1	1
Non-Hindu	8 (23.5)	26(76.5)	0.038^*∗*^	0.41(0.18–0.97)	0.36(0.13–1.03)

Type of school					
Government	47 (17.3)	224(82.7)	—	1	1
Private	3(2.2)	132(97.8)	<0.001^*∗*^	9.23(2.81–30.25)	6.10(1.58–23.46)

Size of family					
1 to 4	6 (5.0)	114(95.0)	—	1	1
5 and above	44 (15.4)	242(84.6)	0.004^*∗*^	0.29(0.12–0.70)	0.45 (0.17–1.20)

Mother's education					
Illiterate	9 (9.4)	87(90.6)	—	1	1
Read and write	19 (29.2)	46(70.8)	—	0.25(0.11–0.60)	0.22(0.07–0.64)
Up to grade 10	16(9.6)	150(90.4)	<0.001^*∗*^	0.97(0.41–2.29)	0.77(0.27–2.24)
SLC and above	6(7.6)	73(92.4)	—	1.26(0.43–3.70)	0.33(0.08–1.32)

Father's education					
Illiterate	13(35.1)	24(64.9)	—	1	1
Read and write	5(12.8)	34(87.2)	<0.001^*∗*^	3.79(1.20–12.0)	3.69(0.81–16.89)
Up to grade 10	27(10.8)	222(89.2)	—	4.43(2.02–9.71)	5.15(1.84–14.39)
SLC and above	5(6.2)	76(93.8)	—	8.23(2.66–25.46)	3.55(0.83–15.15)

Father's occupation					
Agriculture	21(23.9)	67(76.1)	—	1	1
Business and services	12(7.6)	146(92.4)	0.001^*∗*^	3.81(1.78–8.20)	1.48(0.51–4.32)
Wage and foreign labor	17(10.6)	143(89.4)	—	2.64(1.30–5.32)	1.96(0.71–5.45)

Earning status					
No earning	17(35.4)	31(64.6)	<0.001^*∗*^	1	1
Earning	33(9.2)	325(90.8)	—	0.41(0.18–0.97)	2.71(0.99–7.41)

Live with					
Both parents	26(9.8)	239(90.2)	—	1	1
Only with mothers	17(17.9)	78(82.1)	0.018	0.50(0.26–0.97)	0.29(0.12–0.69)
Relatives	7(15.2)	39(84.8)	—	0.61(0.25–1.49)	0.78(0.25–2.42)

^*∗*^Significant at *p* < 0.05, 1 = reference category, ^a^crude odds ratio, ^b^adjusted odds ratio.

## Data Availability

The raw data under identification policy will be provided upon request through an email to the corresponding author.

## Abbreviations

AOR: Adjusted odds ratio
CI: Confidence interval
OR: Odds ratio
SD: Standard deviation
SLC: School leaving certificate
SPSS: Statistical Package for the Social Sciences
M: Municipality
RM: Rural municipality
SMPC: Submetropolitan city.
